# Linear and symmetric synaptic weight update characteristics by controlling filament geometry in oxide/suboxide HfO_x_ bilayer memristive device for neuromorphic computing

**DOI:** 10.1038/s41598-023-36784-z

**Published:** 2023-06-13

**Authors:** Dwipak Prasad Sahu, Kitae Park, Peter Hayoung Chung, Jimin Han, Tae-Sik Yoon

**Affiliations:** 1 Department of Materials Science and Engineering, Ulsan National Institute of Science and Technology, Ulsan, 44919 Republic of Korea; 2 Graduate School of Semiconductor Materials and Devices Engineering, Ulsan National Institute of Science and Technology, Ulsan, 44919 Republic of Korea

**Keywords:** Electronic devices, Electrical and electronic engineering

## Abstract

Memristive devices have been explored as electronic synaptic devices to mimic biological synapses for developing hardware-based neuromorphic computing systems. However, typical oxide memristive devices suffered from abrupt switching between high and low resistance states, which limits access to achieve various conductance states for analog synaptic devices. Here, we proposed an oxide/suboxide hafnium oxide bilayer memristive device by altering oxygen stoichiometry to demonstrate analog filamentary switching behavior. The bilayer device with Ti/HfO_2_/HfO_2−x_(oxygen-deficient)/Pt structure exhibited analog conductance states under a low voltage operation through controlling filament geometry as well as superior retention and endurance characteristics thanks to the robust nature of filament. A narrow cycle-to-cycle and device-to-device distribution were also demonstrated by the filament confinement in a limited region. The different concentrations of oxygen vacancies at each layer played a significant role in switching phenomena, as confirmed through X-ray photoelectron spectroscopy analysis. The analog weight update characteristics were found to strongly depend on the various conditions of voltage pulse parameters including its amplitude, width, and interval time. In particular, linear and symmetric weight updates for accurate learning and pattern recognition could be achieved by adopting incremental step pulse programming (ISPP) operation scheme which rendered a high-resolution dynamic range with linear and symmetry weight updates as a consequence of precisely controlled filament geometry. A two-layer perceptron neural network simulation with HfO_2_/HfO_2−x_ synapses provided an 80% recognition accuracy for handwritten digits. The development of oxide/suboxide hafnium oxide memristive devices has the capacity to drive forward the development of efficient neuromorphic computing systems.

## Introduction

In an ever-evolving world of Internet of Things (IoT) and big data era, the abundant acquisition of digital data, often highly unstructured, needs fast and efficient on-chip data processing for timely decision making. Despite the progress made in conventional Si-based CMOS technology, it still struggles with high computation and energy consumption for data-intensive tasks^1,2^. One of the major concerns is CMOS architecture design where memory and processing units are two separate entities. In addition, the sequential operation processing in such CMOS processors demands constant data transfer between memory and processing unit which caps a limit on computing speed, known as the von Neumann bottleneck^3^. Therefore, a fundamental change in computing hardware and architecture is essential to address the growing demand for data-centric computing operations such as artificial intelligence and machine learning.

The human brain nervous system, consisting of synapses and neurons, is an excellent computing system that processes information in a parallel, event-driven, and distributed structure. Thus, an energy-efficient, high operation speed neuromorphic system is considered as an alternative to the conventional von Neumann system^4^. Neuromorphic computing systems have a highly interconnected network that has inherited salient features of the human brain for parallel and fault-tolerant information processing by mimicking the functionalities of synapses and neurons^5,6^. These synapses can perform both processing and storing information at the same location, thereby reducing energy consumption. Various two- and three-terminal nanoscale electronic memory devices have been extensively studied to emulate the synaptic plasticity behavior, the pattern of learning and forgetting characteristics in the brain^7^. Owing to the simple structure and feasibility of three-dimensional (3D) vertical integration, two-terminal memory devices such as ferroelectric random access memory (FeRAM), phase change random access memory (PCRAM), resistive random access memory (RRAM) and magnetic random access memory (MRAM) have been widely investigated for the application to artificial synapse^8–11^. Among these, RRAM devices, also known as memristive devices, work on the principle of voltage-induced resistance state modulation in a non-volatile manner, and are considered to be suitable candidates for artificial synaptic devices due to low power operation, high scalability, and good CMOS compatibility. A variety of switching materials, including oxides, nitrides, perovskites, and organic materials have been explored for developing RRAM devices^12–18^. Primarily, transition metal oxide-based memory devices such as TiO_2_, NiO_x_, HfO_2_, TaO_x_, ZnO have been widely studied^19–23^.

One of the major challenges in these devices is non-uniformity in switching parameters which arises from the stochastic nature of filament growth dynamics in the switching layer^10^. The critical requirement of an ideal synaptic device is the modulation of a large number of analog conductance states with linear and symmetric change or weight updates to minimize error and achieve high learning accuracy in a neural network^1^. However, in reality, memristive devices show mostly digital switching with non-linear and asymmetric weight updates when they operate by the formation and rupture of conducting filament. Also, memristive devices operating with resistance change originated by redistributed defects suffer from non-uniformity and unreliable switching characteristics due to uncontrolled ion transport through the defects in the switching layer. This leads to temporal (cycle-to-cycle) and spatial (device-to-device) variations in these synaptic devices which adversely affect the computing performances^10^. In memristive devices using filament for conductance modulation, therefore, various approaches to address the randomness of filament formation have been adopted such as impurity doping^24^, metal nanoparticle incorporation^25^, and inserting a bilayer^26^ to confine filaments. In recent times, HfO_2_-based memristors have been explored for synaptic application owing to their scalability and compatibility with the current CMOS technology^24–27^. The switching mechanism in HfO_2_ memristors is broadly attributed to the formation and rupture of nanoscale conductive filaments consisting of oxygen vacancies through redox reaction, ion migration or nucleation process.

Furthermore, bilayer structured HfO_2_ memristors have been reported with improved electrical performances compared to single-layer oxide devices. For example, Ye et al. reported lower operation voltage and improved uniformity of HfO_2_/TiO_2_ bilayer structured memristors compared to single-layer devices^28^. Kim et al. exhibited gradual conductance change in HfO_x_/AlO_y_ devices and demonstrated biological synaptic characteristics even at elevated temperatures confirming the stability and sustainability of neuromorphic chips^29^. In addition, a stable two-level resistive switching feature was reported on Pt/HfO_2_/HfO_2−x_/TiN devices which was explained based on the migration of oxygen ions by utilizing TiN electrode as an oxygen reservoir^30^.

Besides filamentary switching memristors, interface-type switching devices have also been reported to show highly uniform switching characteristics due to homogeneous change of resistance state through the interface reaction. Thus, the conductance of the device could gradually change, avoiding any abrupt change and requirement of electroforming process, which is one of the important properties of artificial synapses^31,32^. Hansen et al. has reported area-dependent switching in oxide heterojunction memristors using Al_2_O_3_/Nb_*x*_O_y_ double-barrier layer with uniform current distribution for high and low resistance states; yet the device retention time significantly affected, which is critical to guarantee pattern classification accuracy^33^. Kunwar et al. demonstrated versatile synaptic functions with an excellent uniformity through interface-controlled Au/Nb-doped SrTiO_3_ Schottky structure with reliable retention^34^. A two-terminal charge trapped memristor based on Pt/Ta_2_O_5_/Nb_2_O_5-x_/Al_2_O_3-y_/Ti device has been reported exhibiting highly self-rectifying and nonlinear characteristics with a long retention time achieving a good pattern recognition challenge^35^.

In the memristors with resistance change using the formation of filament consisting of oxygen vacancies, the sequential steps of creation of oxygen vacancies during an initial forming process, and its drift and diffusion in the oxide layer lead to switching in the device. Thus, considering the improved memory and synaptic properties in heterojunction bilayer HfO_2_ devices with different oxide materials, it is of great interest to explore the switching property and synaptic behavior of the device in a homojunction bilayer HfO_2_ structure. Compared with the heterojunction bilayer devices composed of different oxide layers, the homojunction bilayer devices employing the same constituent oxide layers are expected to have more reliable and controllable switching behaviors because the creation and redistribution of oxygen vacancies would occur within the homojunction layers. Typically, creation of oxygen vacancies during an initial forming process, and its drift and diffusion in the oxide layer leads to switching in the device. However, in the devices with a stoichiometric oxide layer, it is difficult to control the electroforming process due to a low vacancy concentration at the initial point and thus requires a large voltage for electrical breakdown which may lead to large conductance change. Therefore, to ease the need for creating oxygen vacancies, an oxide/suboxide structure can be considered for reliable switching performances^36^. Thus, in this work, we developed a homojunction oxide/suboxide HfO_x_ bilayer memristive device, i.e., Ti/HfO_2_/HfO_2−x_/Pt, to emulate various synaptic functions. The oxide/suboxide HfO_x_ bilayers are deposited using atomic layer deposition (ALD) system under different deposition temperature to control the stoichiometry of oxide layers. The top HfO_2_ and bottom HfO_2−x_ layers served as a stoichiometric resistive (oxygen vacancy-poor) layer and a relatively conductive (oxygen-deficient or oxygen vacancy-rich) layer, respectively. Therefore, the concentration gradient of oxygen vacancies plays a significant role in the device operation of oxide bilayer device. The fabricated synaptic device could operate at low voltage with stable memory characteristics by limiting the location of filament formation and rupture in the upper layer of HfO_2_ switching matrix. In particular, the tunable pulse operations such as incremental step pulse programming (ISPP) were employed by varying pulse amplitude, step, and width, then enhanced synaptic characteristics such as linear and symmetric weight update along with basic synaptic learning functions of biological brains were demonstrated. These results may pave the way for designing an effective artificial synaptic device for memory and neuromorphic computing.

## Results and discussion

**Figure 1:**
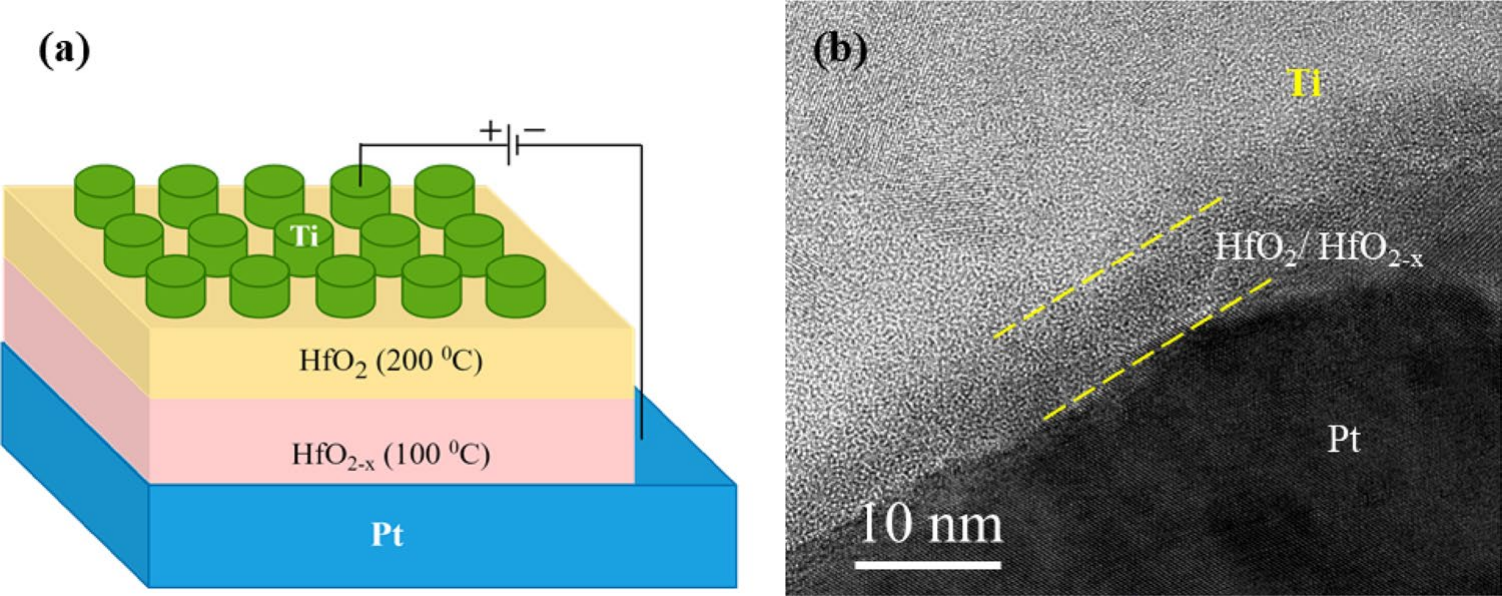
(**a**) Schematic diagram of Ti/HfO_2_/HfO_2−x_/Pt memristive device and (**b**) cross-sectional high-resolution TEM micrograph of the bilayer device.

Figure 1a depicts the schematic diagram of the device with oxide and suboxide hafnium oxide bilayer with different stoichiometry, i.e., Ti/HfO_2_/HfO_2−x_/Pt. From the cross-sectional high-resolution TEM micrographs in Fig. 1b, the switching oxide bilayer is found to be the mixture of crystalline and amorphous phases with total thickness of ~ 6 nm. In order to make a clear distinction of HfO_2_/HfO_2−x_ bilayer structure, it was also deposited on the flat SiO_2_ substrate as shown in Fig. S1 (supplementary information). The bilayer has a uniform and flat interface with top Ti layer. In addition, a crystalline grain is also observed, which is well coincident with our previous reports^37^. Notably, the interface between HfO_2_ and HfO_2−x_ is not clearly observed in the micrograph unlike the typical heterojunction bilayer structures. It should be noted that the stoichiometry, and oxygen vacancy concentration of a stoichiometric HfO_2_ and oxygen-deficient HfO_2−x_ layer, and the resistive switching characteristics with those layers are quite different, as will be further discussed later. Nevertheless, the microstructure in the bilayer turned out to be homogeneous, which is beneficial to induce the creation and redistribution of oxygen vacancies for the resistive switching.

**Figure 2:**
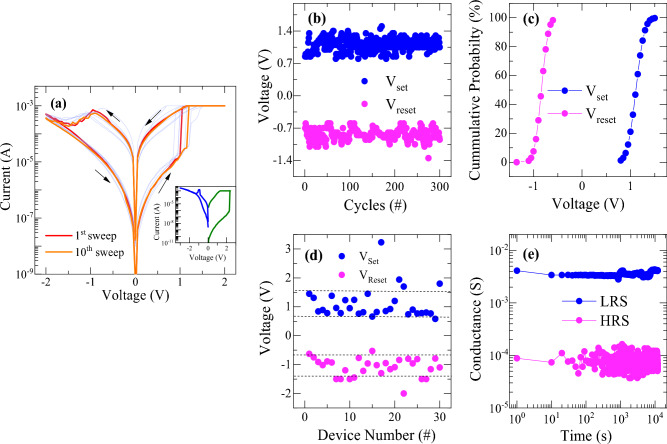
(**a**) Typical *I*–*V* characteristic curves of the memristive device at the maximum voltage sweep range of ± 2 V with voltage scanning rate of 50 mV/s (inset: *I*−*V* curve for initial electroforming process), (**b**) cycle-to-cycle variation of set and reset voltage for 300 sweeps, (**c**) cumulative probability distribution of set and reset voltage indicating a narrow distribution of parameters, (**d**) device-to-device variation of switching voltages, and (**e**) retention characteristics of LRS and HRS states for more than 10^4^ s.

The typical current–voltage (*I*−*V*) characteristic curves of HfO_2_/HfO_2−x_ bilayer memristive device were obtained using DC voltage sweep measurements, as shown in Fig. 2a. The device was in high resistance in its pristine state and required a soft dielectric breakdown with a limit to compliance current, called electroforming process. The device underwent the initial electroforming process by applying a voltage sweep up to + 2.4 V with a compliance current of 1 mA as shown in the inset of Fig. 2a. The voltage scanning rate was 50 mV/s. An abrupt change in current to maximum value was observed at ~ 2.3 V transitioning to a low resistance state (LRS). Again, the device was retraced back to its high resistance state (HRS) by applying a voltage of − 2.5 V. After the electroforming process, the device could be reversibly altered between LRS and HRS states consistently, exhibiting a typical bipolar resistive set and reset switching behaviors. Figure 2b shows cycle-to-cycle variation of switching voltages for 300 sweeping cycles, whose *I*−*V* sweep curves are shown in Fig. S2 (supplementary information). The set voltage for switching from HRS to LRS is found to be within 0.87 to 1.5 V and reset voltage for switching from LRS to HRS is nearly − 0.62 to − 1.1 V. It indicates a narrow distribution of switching voltages, which is plotted as cumulative probability distribution of switching voltage in Fig. 2c. The statistical analysis of switching voltages revealed that the coefficient of variation for set and reset voltage (standard deviation (σ) to mean values (µ)) is found to be 11.8% and 13.22%, respectively. Additionally, the device-to-device variation of switching voltages was also depicted in Fig. 2d. The set and reset voltage values measured at 30 randomly selected devices remain within a range from 0.58 to 1.45 V and − 0.53 to − 1.5 V, respectively, for most of the devices. This verifies a low voltage operation, good reliability and reproducibility of switching performance of the devices. Figure 2e shows the retention property in both HRS and LRS states. There is no significant degradation in the conductance of the states were observed for more than 10^4^ s thanks to the robust nature of filamentary switching. The stability of the device was also verified by performing an endurance test under pulse measurement at a read voltage of 0.5 V with a 60 µs pulse indicating more than 4000 pulse cycles, as shown in Fig. S3.

In Fig. 2, the memristive device exhibits only two conductance states with a quick transition between HRS and LRS due to filamentary switching behavior when DC voltage sweep measurements were performed with the voltage scanning rate of 50 mV/s. However, a progressive conductance change with multilevel resistance state modulation needs to be achieved to demonstrate the synaptic characteristics. In view of this, it is essential that the present devices enable to fine tune the various conductance states by controlling switching operation parameters such as limiting the compliance current or controlling set and reset voltage conditions.

**Figure 3:**
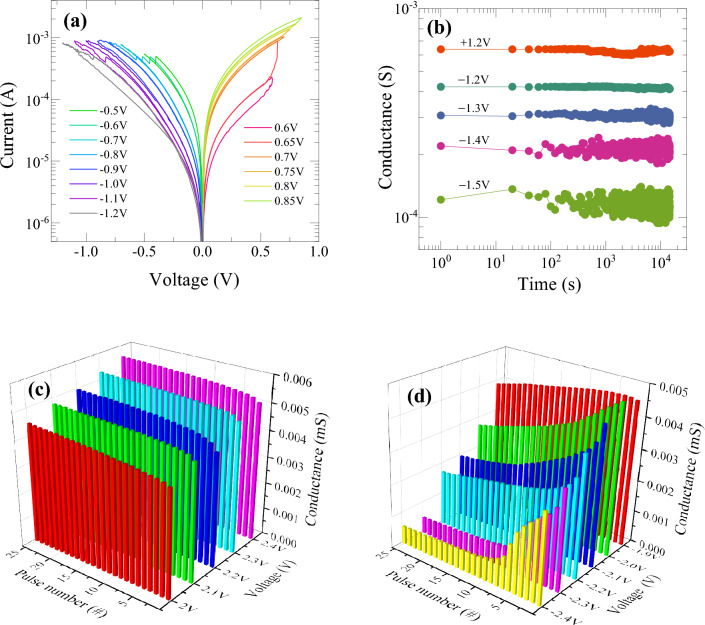
(**a**) *I*−*V* characteristic curves of the memristive device as varying maximum sweep voltage with the reduced voltage scanning rate of 5 mV/s, (**b**) retention of multilevel conductance states, and multiple conductance change in the pulse operation for (**c**) set and (**d**) reset switching process.

In the present devices, the analog switching could be achieved with multiple conductance states by controlling the voltage sweep scanning rate. Note that the analog conductance modulation also requires a pre-forming process to initiate the switching. As shown in Fig. 3a, the device can be tuned to achieve various analog conductance states by continuously increasing the amplitudes of set and reset voltages. To achieve the gradual switching, a lower voltage scanning rate of 5 mV/s was applied to the device without any restriction on compliance current. The device exhibits distinguishable increasing conductance states as the amplitude of voltage is increased from 0.6 to 0.85 V, indicating that the device could be able to mimic the potentiation process of biological synapses as will be confirmed also by voltage pulse operations. Similarly, a decrease in current is observed as the magnitude of negative bias voltage is increased which demonstrates the potential to replicate the depression of the biological synapse. Furthermore, it is clear that compared to positive bias sweep, a greater number of conductance states can be achieved by controlling reset stop voltages. This is an obvious effect of filamentary switching behavior that causes a prominent increase in current due to generation of higher concentration of oxygen vacancy and thus restricts further increasing in current which limits the number of states at the positive bias sweep. On the other hand, due to gradual nature of filament rupture during negative bias sweep, a large number of states can be observed. The retention performance of these different conductance states was also examined with a pulse width of 100 µs at a read voltage of 0.5 V, as shown in Fig. 3b. The LRS state at + 1.2 V and four different HRS states at negative biases show no degradation of current, indicating reliability of the device. The superior retention properties come from the use of resistance change by robust filamentary switching compared to non-filamentary resistance change. However, it can be observed that the device shows more variation as we apply higher negative reset voltage to turn to high resistance. This is obvious because of low current measurement which is consequence of random motion of electrical charge carriers and diffusion of oxygen ions in the device. Additionally, the stochastic nature of the switching process may also introduce variability in the time required for the filaments to rupture, further complicating the measurement and control of the device's electrical properties. Nevertheless, the analog switching behavior with gradual conductance modulation of multilevel states and stable retentivity of each state makes it suitable for demonstrating reliable synaptic plasticity. The results of multilevel conductance states simply by reducing DC voltage scanning rate indicate that the conductance states could be modulated in an analog manner by controlling the voltage application conditions, especially in pulse applications as demonstrated in following results.

For artificial synapse device application with synaptic behaviors of the present memristive devices, it is essential to demonstrate the synaptic weight update characteristics with respect to pulse voltage rather than DC voltage sweep. We have examined the conductance change, corresponding to synaptic weight update, in response to the application of both positive and negative voltage pulses of 10 µs width and pulse interval time of 1 µs at a readout voltage of 0.2 V. As shown in Fig. 3c and d, the conductance of each state increases (decreases) as the amplitude of positive (negative) voltage pulse increases along with the pulse number. There is a quite similar behavior between DC sweeping mode and pulse mode in positive bias condition, where the increase in conductance for different amplitude of voltage is not significant. It is assigned to filamentary nature of current conduction in the devices. Due to availability of a large amount of oxygen vacancies, the current increases rapidly for the very first pulse; thus, subsequent increase in current is limited. However, due to gradual rupture of filament in negative bias, current decreases sequentially as depending systematically on the voltage amplitude and number of pulses. Obviously, the conductance decreases more significantly at the first few pulses with the higher voltage amplitude and consequently saturates. On the other hand, at the lower amplitude, e.g., − 1.9 and − 2.0 V, the conductance is found to decrease gradually. These characteristics verify the possibility of fine conductance tuning to improve synaptic weight update by optimizing pulse application conditions as discussed in the Fig. 8, 9, 10, 11.

**Figure 4:**
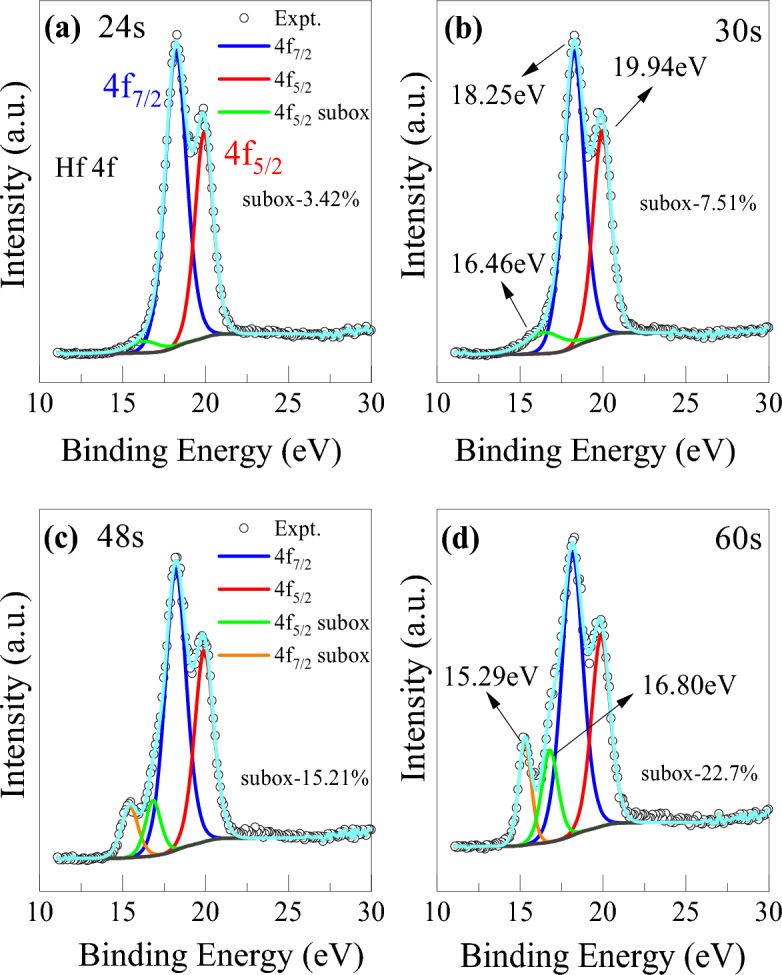
XPS spectra of Hf 4f peak evolution with etching time of (**a**) 24, (**b**) 30, (**c**) 48, and (**d**) 60 s from the top surface in bilayer HfO_2_/HfO_2−x_ structure.

The physical origin of switching characteristics in this bilayer device may come from different concentrations of oxygen vacancies in each layer, which contribute to the gradual change of filament geometry for analog synaptic weight update characteristics. To understand the composition and chemical bonding state of the bilayer structure, XPS core level and depth analyses were performed on the devices. All the peaks were fitted with Gaussian–Lorentzian (G-L) functions after a Shirley background subtraction. Figure 4 illustrates the core level spectra of Hf 4f peak in the HfO_2_/HfO_2−x_ bilayers on Pt bottom electrode with Ti adhesion layer at different etching times from top oxide layer to bottom Pt layer. From the XPS depth profile of the bilayer structure, the positions of 24 and 30 s are within the stoichiometric upper HfO_2_ layer and the ones of 48 and 60 s are within an oxygen-deficient lower HfO_2−x_ layer as shown in Fig. 6. The Hf 4f spectra are deconvoluted into two spin–orbit split peaks of binding energies 18.25 eV and 19.94 eV corresponding to Hf 4f_7/2_ and Hf 4f_5/2_ peaks, respectively, which are assigned to Hf–O bonding (Hf^4+^) from stoichiometric HfO_2_ layer^38^. Additionally, a doublet peak located at lower binding energies of 15.29 eV and 16.80 eV is attributed to low chemical valence states of Hf^n+^-O (n < 4)^39^. These two weaker peaks indicate the suboxides of hafnium valence states in the HfO_2−x_ layer, which suggests the presence of abundant oxygen vacancies. The content of two strong Hf peaks are roughly calculated by evaluating the area under each peak and it comes out that the percentage of Hf^4+^and Hf^n+^ is 96.58% and 3.42% respectively at 24 s etching time. Also, it is clear that the percentage of suboxide Hf^n+^ increases with etching time from 3.42 to 22.7% hinting that the concentration of oxygen vacancies is higher at the lower HfO_2−x_ layer, due to its lower deposition temperature in ALD process. A complete Hf spectra deconvolution starting from the etching time of 0 to 90 s have been shown in Fig. S4 (supplementary information).

**Figure 5:**
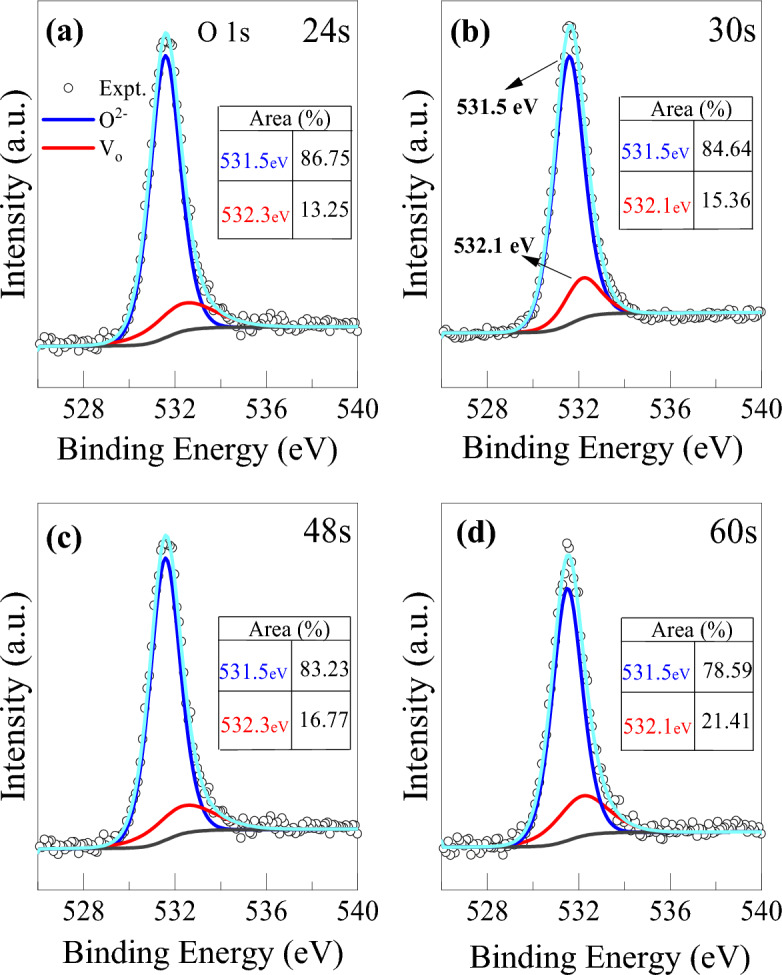
XPS spectra of O 1s peak evolution with etching time of (**a**) 24, (**b**) 30, (**c**) 48, and (**d**) 60 s from the top surface in bilayer HfO_2_/HfO_2−x_ structure (inset table: percentage of area under each peak of two binding energies).

Figure 5 represents the O 1s core level spectra of the bilayer at different etching times. The O 1s spectra are deconvoluted into two peaks of binding energies at 531.5 eV and 532.3 eV which are assigned to lattice oxygen and non-lattice oxygen with oxygen vacancies, respectively. A minimal shift of O 1s strong peak to lower binding energy with increasing depth from the top surface is observed which may be related to the change in film composition. Alternatively, the peak shift is thought to be modulated by the relative amount of metal ions bonded to oxygens^40^. A higher amount of non-lattice oxygen content is observed to exist in the lower suboxide layer (HfO_2−x_) than in the upper stoichiometric HfO_2_ layer. The percentage of non-lattice oxygen ions increases to 21.41% at the lower oxygen-deficient HfO_2−x_ layer after 60 s of etching time, while it is 13.25% at the upper stoichiometric HfO_2_ layer. A complete O 1s spectra deconvolution from 0 to 90 s of etching have also been presented in Fig. S5 (supplementary information). The O 1s profiles disclose consistently the graded oxygen concentration in the bilayer structure as same with Hf 4f profiles.

**Figure 6:**
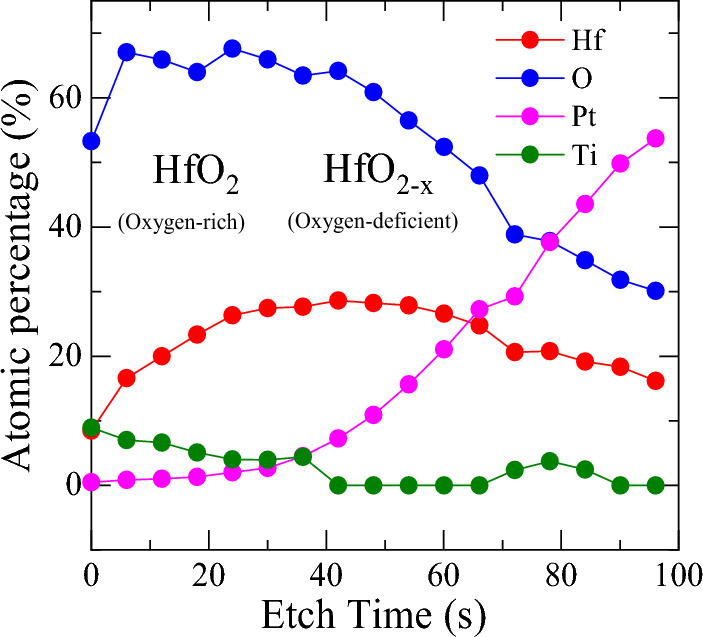
XPS depth profile of bilayer HfO_2_/HfO_2−x_ structure.

Figure 6 shows the composition depth profile obtained from XPS analysis. The upper layer at shorter etching time has the stoichiometry close to HfO_2_, while the lower layer region at longer etching time has reduced oxygen content. In addition, the oxygen content keeps decreasing in the lower oxygen-deficient HfO_2−x_ layers. From the Hf 4f and O 1s core level spectra and the composition depth profile, it is conclusive that the bilayer structure is composed of the upper stoichiometric HfO_2_ and the lower oxygen-deficient HfO_2−x_ layers.

**Figure 7:**
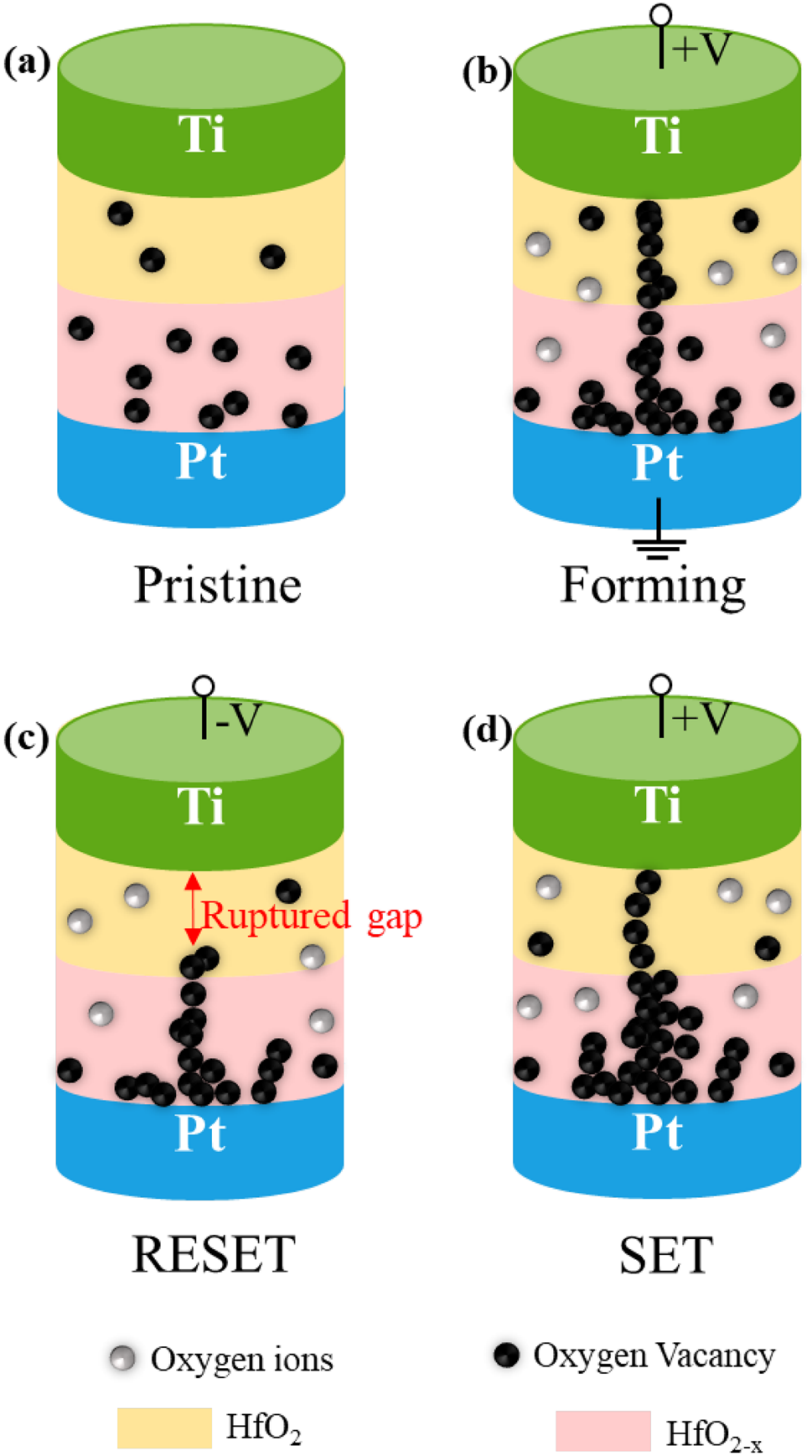
Schematic illustration of resistive switching mechanism in Ti/HfO_2_/HfO_2−x_/Pt memristive devices.

Generally, the concentration gradient of oxygen vacancies plays a major role in the filament-based resistive switching device. The XPS results also confirmed the presence of abundant oxygen vacancies in as-prepared devices. However, a mere increase in oxygen vacancy concentration is not an effective way for stable switching performance, but its effective distribution is key to uniform switching characteristics. Because the present device has the homojunction bilayer structure of HfO_2_/HfO_2−x_ with different oxygen content in each layer, as confirmed from XPS analysis, its resistive switching by filament formation would be determined by the redistribution within the bilayer as schematically illustrated in Fig. 7. The as-fabricated device in its pristine state has random oxygen vacancies more in the bottom HfO_2−x_ layer than top HfO_2_ layer (Fig. 7a). During the electroforming process upon applying a positive bias voltage to the top Ti electrode, a few amounts of oxygen vacancies are more generated and the pre-existing randomly distributed oxygen vacancies in the lower suboxide (HfO_2−x_) layer tend to align to participate in the conductive filament formation under the external electric field (Fig. 7b). This results in the transition from an initial HRS with 10^9^ Ω to LRS with 2000 Ω soon after filament formation indicating a possible migration of oxygen vacancies from the lower suboxide layer to upper oxide (HfO_2_) layer. In contrast to the single layer device where oxygen vacancies are generated inside the single layer through the process of oxygen migration from oxide to top electrode (e.g., Ti), the present bilayer device enables the redistribution of oxygen vacancies through exchanging between the two layers. Therefore, it would have a more stable resistance change, particularly for gradual change in an analog manner. Generally, a wider filament will grow in the lower suboxide layer compared to the upper oxide layer due to the availability of higher oxygen vacancies in the lower oxygen-deficient suboxide layer, and a confined filament will grow inside the upper oxide layer.^41^ This minimization of filament growth location in the upper oxide layer brings more stable and reproducible switching performances in the bilayer device compared to the single layer devices. A negative bias takes the device to reset process by rupturing filament partially at the upper oxide layer while the major part of filament remains intact in the lower suboxide layer thanks to its higher oxygen vacancy concentration (Fig. 7c). During reset process, the conductive filament starts rupturing from the tip of the top electrode as a result of the recombination between oxygen ions and oxygen vacancies, which leaves an insulating gap. Subsequently, when a positive bias is applied, the device sets to LRS again by reconnecting the filament. However, the set voltage required to achieve LRS is lower than initial forming voltage due to reconnection of only a fraction of pre-existing filament (Fig. 7d). Therefore, the utilization of an oxide/suboxide bilayer structure leads to confinement of filament location which narrows the distribution of set and reset voltages (Fig. 2b and d). The use of the redistribution of oxygen vacancies within the bilayer structure provides the potential of precise change of filament geometry, which is crucial for realizing analog resistance change to mimic analog synaptic weight update as shown in following results.

**Figure 8:**
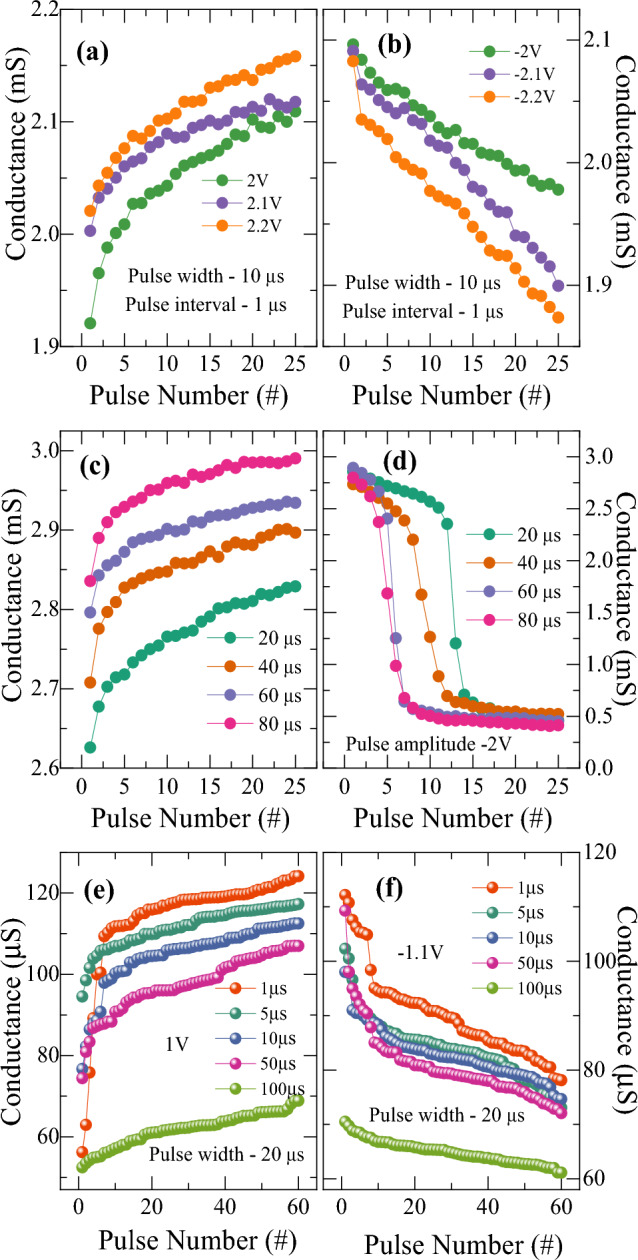
Conductance modulation characteristics under various pulse schemes for potentiation and depression as varying (**a**) and (**b**) pulse amplitude, (**c**) and (**d**) pulse width, and (**e**) and (**f**) pulse interval time.

In the next section, we have demonstrated the various synaptic functions in terms of potentiation and depression in Ti/HfO_2_/HfO_2−x_/Pt memristive device at different voltage pulse application conditions. As the memristive conductance closely follows with biological synaptic weight, the change of conductance states corresponds to the synaptic weight update for the learning process with long term potentiation (LTP) and long-term depression (LTD) of the biological synapse. As shown in Fig. 8, the synaptic weight of the device depends on various stimulation parameters of input pulse including its amplitude, width and interval time between pulses. To emulate the biological synaptic characteristics, several other works have also considered various programming schemes such as use of incremental pulse amplitude and pulse width^31,42–44^, addition of a heating spike before set and reset pulses^45^, current pulse mode^46^ and device processing approaches such as N_2_ annealing treatment^47^. Figure 8a shows a monotonously increasing conductance (potentiation) with the number of pulses by applying identical square pulse waveform of amplitude of 2 V with a width of 10 µs and pulse interval of 1 µs. A constant readout voltage of 0.5 V is applied to read the conductance after each potentiation pulse. Applying a higher amplitude pulse induces the migration of more oxygen vacancies leading to an increased conductance. Similarly, a gradual decrease in conductance (depression) is observed with opposite pulse spikes of same conditions as shown in Fig. 8b. Furthermore, it is observed that the conductance increases or decreases steadily with increasing the pulse amplitude, which is also consistent with the results of DC sweep measurement by varying amplitude of sweep voltages. The potentiation and depression behavior can be explained based on the continuous modulation of conductance in a confined region of filament with its dimensional change by a small amount of oxygen vacancies. The increase of pulse amplitude controls the dimension of filament which conceives to potentiation and depression behavior with variable update ranges.

Figure 8c and d show pulse width-dependent synaptic weight modulation at a fixed pulse amplitude of ± 2 V, pulse interval of 1 µs and readout at 0.5 V. The conductance of the device increases with the pulse number as the pulse width is increased from 20 to 80 µs. It is also noticed that a larger pulse width requires a smaller number of pulses to induce conductance increment and vice-versa. This could be due to that applying longer pulse provides oxygen vacancies with more sufficient time to migrate for the conductance change. In particular for the depression behavior, Fig. 8d shows the gradual decrease of conductance for a shorter pulse width while a sharp decrease for longer pulse width. For example, 12 pulses are required to reset the conductance for a pulse width of 20 µs whereas it needs only 4 pulses for pulse width of 80 µs. Also, the conductance decrease at pulse width of 20 µs is quite abrupt, compared with the gradual decrease at 10 µs pulse width shown in Fig. 8b, disclosing the strong dependence also on the pulse width.

The dependence of synaptic weight update on pulse interval time was also examined at the condition of a constant pulse amplitude of 1 V and − 1.1 V with a pulse width of 20 µs. As shown in Fig. 8e and f, the conductance change is found to decrease for both potentiation and depression behaviors as the pulse interval time is increased from 1 to 100 µs. It is noticeable that the conductance changes are rather significant at the first few pulses and then saturate with the increase of pulse number for the pulse interval time up to 50 µs. However, the conductance change is fairly gradual with almost identical increments with respect to the pulse number for a larger pulse interval time of 100 µs. The results showing the dependence of conductance change on pulse interval time indicate that it takes time for the filament geometry to be stabilized. Thus, the longer interval time results in more gradual and stabilized conductance changes. It is rational that the conductance change depends on pulse interval time between adjacent pulses because it involves the generation, recombination, and migration of oxygen vacancies to adjust the filament geometry. It implies that some unstable species undergo time-dependent transient dynamics in either generation, recombination, or migration. For example, unstable oxygen vacancies would disappear over time by recombining with oxygen ions, thus they do not contribute to the conductance change in the condition of longer pulse interval time. Therefore, the oxygen vacancies generation, recombination, and migration driven by electric field and the energy required for conductance change in the memristive device are well affected by the pulse conditions such as pulse amplitude, pulse width and pulse interval time.

In the artificial synapse device application, the linear and symmetric synaptic weight update characteristics as well as its excellent endurance properties are crucial for energy-efficient and accurate learning operations^48,49^. In general, the dynamics of conductance change becomes non-linear and asymmetric in nature due to abrupt set and reset transition in filamentary synaptic devices. For instance, the repeated measurements of potentiation and depression behaviors read at 0.5 V using identical pulse amplitude of ± 1.5 V with a width of 10 µs and interval of 100 µs disclose an excellent endurance during repeated cycles of more than 3000 pulses without significant degradation as shown in Fig. S6. To calculate the linearity of weight update during the potentiation and depression processes, the conductance change of potentiation and depression with the number of pulses can be modeled by the following equations in MATLAB R2023a software^50^,

1 $$G_{{{\text{potentiation}}}} = {\text{B}}\left\{ {1 - exp\left( { - \frac{P}{A}} \right)} \right\} + G_{{{\text{min}}}}$$

2 $$G_{{{\text{depression}}}} = - {\text{B}}\left\{ {1 - exp\left( { - \frac{P}{A}} \right)} \right\} + G_{{{\text{max}}}}$$

3 $${\text{B}} = { }\frac{{G_{{{\text{max}}}} - G_{{{\text{min}}}} }}{{1 - exp\left( { - \frac{{P_{max} }}{A}} \right)}}$$

where $G_{{{\text{potentiation}}}}$ and $G_{{{\text{depression}}}}$ are the conductance for potentiation and depression, respectively. $G_{{{\text{max}}}} , G_{{{\text{min}}}}$ and $P_{max}$ are directly extracted from the experimental data, which represents the maximum conductance, minimum conductance and the maximum pulse number required to switch the device between the minimum and maximum conductance states. *A* is the parameter that controls the nonlinear behavior of weight update. *B* is a function of *A* that fits the functions within the range of $G_{{{\text{max}}}} , G_{{{\text{min}}}}$ and $P_{max}$.

The non-linearity values for potentiation and depression are calculated to be 4.88 and 3.7 respectively for identical pulse scheme as shown in Fig. S6(a). The synaptic weight update is fairly non-linear and asymmetric. The potentiation current increases abruptly and then saturate more quickly than depression current following an obvious nature of filamentary switching as shown in Fig. S6(b).

**Figure 9:**
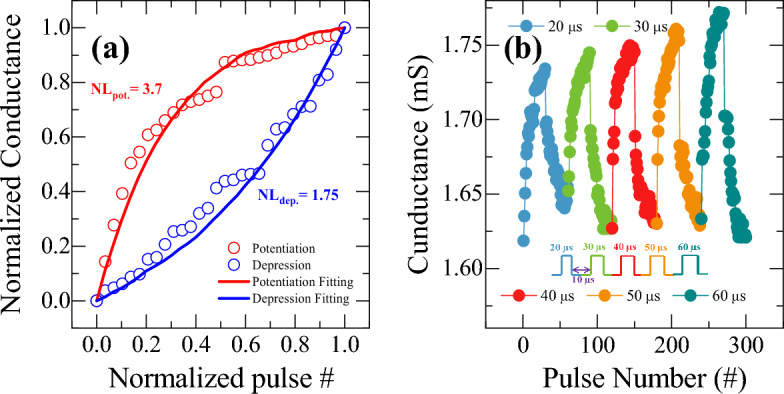
(**a**) Non-linearity of synaptic weight update from Ti/HfO_2_/HfO_2−x_/Pt bilayer memristor under different pulse widths. (**b**) Repetitive conductance modulation by varying pulse width from 20 to 60 µs with a pulse amplitude of + 1.2 and − 1.3 V for potentiation and depression, respectively, with a pulse interval time of 10 µs.

**Figure 10:**
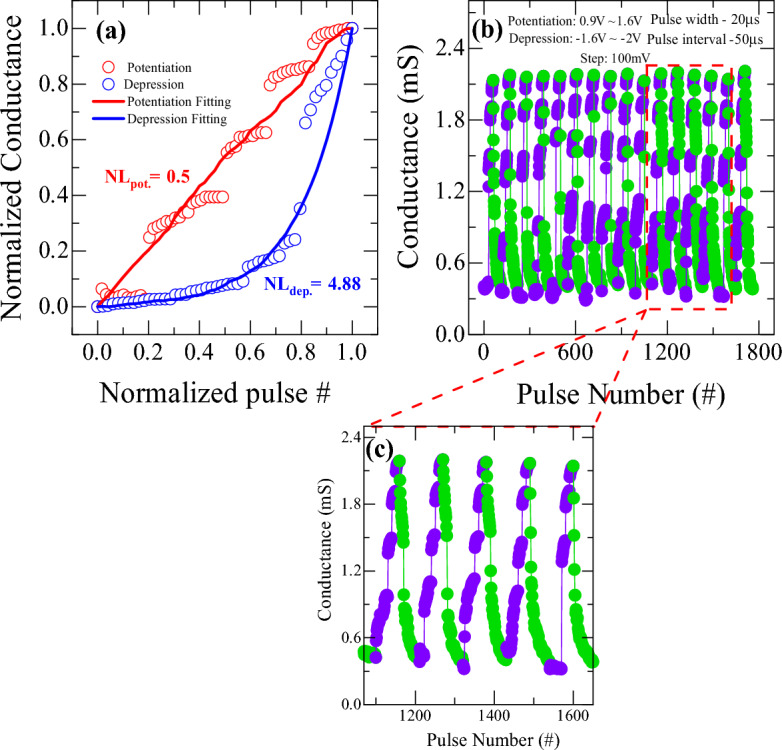
(**a**) Non-linearity of synaptic weight update from Ti/HfO_2_/HfO_2−x_/Pt bilayer memristor by using non-identical pulse spikes under incremental step pulse programming (ISPP) operation. (**b**) Conductance modulation by using non-identical pulse spikes ISPP operation scheme with a constant step voltage of 100 mV. (**c**) An enlarged view of (**b**).

**Figure 11:**
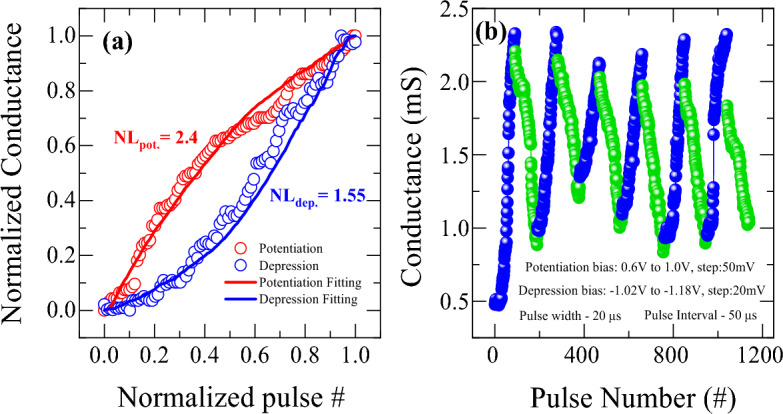
(**a**) Improved non-linearity of synaptic weight update of bilayer memristor device by using ISPP mode with optimized pulse condition. (**b**) Conductance modulation by using non-identical pulse spikes under ISPP operation scheme with optimized step voltage.

To improve the linearity and symmetry of weight update for potentiation and depression, various pulse operation conditions were employed. Figure 9 shows the potentiation and depression behavior as varying the pulse width conditions. The device shows an improved linearity and symmetry of weight update when operates with variable pulse width as compared to previous case of constant pulse width. In this measurement, the potentiation and depression pulse amplitude are kept constant at + 1.2 and − 1.3 V, respectively, with the constant pulse interval of 10 µs. It is also observed that the potentiation conductance increases and depression conductance decreases systematically with the increase of pulse width, which follows with behavior shown in Fig. 8c and d. The non-linearity values for potentiation and depression are found to be 3.7 and 2.03 respectively as shown in Fig. 9a. Although the linearity and symmetry seem to be improved, increasing pulse width could not sufficiently increase the conductance dynamic range as shown in Fig. 9b, which is also an essential condition for improving the pattern recognition accuracy in neuromorphic computing with high resolution. Therefore, to address the dynamic range with high resolution, linearity, and symmetry of synaptic weight update, an incremental step pulse programming (ISPP) operation scheme was adopted, which has been applied to programming in non-volatile flash memory^51^. Non-identical pulse operation programming achieves faster switching times, improved endurance, and enhanced scalability in memory devices. However, such a complex programming scheme requires non-trivial design efforts from the peripheral circuit's perspective because it requires precise calibration of the pulse amplitudes and pulse widths for each memory cell based on its current conductance state before applying the programming pulses. The calibration process requires additional circuitry which can increase the complexity and cost of the memory device. Figure 10 shows the potentiation and depression behaviors as applying non-identical pulses whose amplitude increases after every 10 pulses in steps of 100 mV from 0.9 to 1.6 V with a constant pulse width of 20 µs. The synaptic weight in the potentiation and depression can be modulated by up to 4 times of initial resistance value. Figure 10a shows non-linearity curve with normalized conductance modulation for non-identical pulse operation. Although the non-identical ISPP operation scheme improved non-linearity of potentiation to 0.5, it fails to improve depression non-linearity (4.88). However, an abrupt change of conductance during update is still observed due to filamentary nature of conductance change, as shown in Fig. 10b. An enlarged view of the abrupt transition was shown in Fig. 10c for better representation. This abrupt change could be eliminated further by properly controlling step voltage and much improved gradual weight update could be achieved, as shown in Fig. 11. An optimized pulse schemes using, non-identical pulses with a pulse width of 20 µs and pulse interval of 50 µs in reduced steps of 50 mV from 0.6 to 1 V for potentiation and step voltage of 20 mV from − 1.02 to − 1.18 V for depression was adopted. This resulted in a significant improvement in non-linearity parameters for both potentiation and depression to 2.4 and 1.55 respectively, as shown in Fig. 11a. As illustrated in Fig. 11b, an almost complete linear and symmetric weight update could be achieved through precisely controlled change of filament geometry.

Some of the recent published state of the art work based on HfO_2_ bilayer synaptic devices is compared with the present work and summarized in Table 1.

**Table 1:** Performance comparison of switching parameters of various HfO_x_ bilayer synaptic devices.

Device	Set/reset voltage	Endurance	Retention (sec)	On/Off ratio	Non-linearity	References
Au/HfO_x_/HfO_2_/Pt	+ 0.2 V/− 0.18 V	50	10^4^	> 10	NA	Ref.^52^
Pt/HfO_2_/HfO_*x*_/TiN	− 1.6 V/+ 1.1 V	10^4^	10^5^	10^3^	NA	Ref.^53^
Ti/HfO_x_/AlO_y_/TiN	+ 1 V/− 1.4 V		10^4^	~ 10	1.06/5.43	Ref.^54^
ITO/ZnO/HfO_x_/ITO	+ 1.8 V/− 2 V	10^3^	10^4^	~ 10^2^	3.19/2.4	Ref.^55^
TiN/Ti/HfO_x_/TaO_y_/HfO_x_/Au	− 5 V/+ 5 V	10^6^	10^5^	50	5.3/11.9	Ref.^56^
Ti/HfO_2_/HfO_2−x_/Pt	+ 1.1 V/− 0.84 V (DC measurement)	300	> 10^4^	> 10^2^	2.4/1.55	This work
	+ 1 V/− 1.2 V (pulse measurement)	4 × 10^3^	> 10^4^	~ 20		

**Figure 12:**
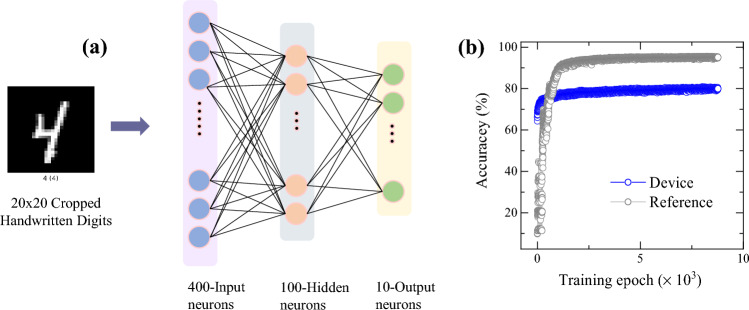
(**a**) Schematic of three-layer perceptron based neural network used for MNIST pattern recognition and (**b**) learning accuracy rate with training epochs using stochastic gradient descent (SGD) algorithm.

In order to evaluate the potential applicability and performance of the present bilayer synaptic device for neuromorphic hardware implementation, the pattern recognition accuracy was examined by the simulation using an artificial neural network system, NeuroSim + , with the normalized conductance values extracted from the potentiation/depression cycle of bilayer synaptic device. More specifically, a three-layer multilayer perceptron (MLP) neural network was used which consists of 400 neurons as input layers, 100 hidden neurons and 10 output neurons corresponding to 10 classes of digits (0–9) as shown in Fig. 12a. A cropped 20 × 20 pixels image from the MNIST data set was used here as an input for the image recognition task and each pixel from the image corresponds to one neuron of the input layer. Figure 12b shows the learning accuracy of the device acquired from the MLP as a function of the number of training epochs. For each epoch, 60,000 training data sets and 10,000 test data sets were used to evaluate the recognition accuracy. The MLP neural network simulator uses a stochastic gradient descent method to update weights which calculates the error from current weight values and then propagates in backward to adjust the weight so as to minimize the prediction error^57^. We also considered various non-ideal parameters including nonlinearity, conductance level and variation during training. The baseline reference was obtained by changing the non-linearity value of both potentiation and depression to 1, while maintaining all other inputs such as conductance values, pulse number and pulse voltage conditions. The simulation results show a learning accuracy of ~ 80% which could be further enhanced by improving the switching dynamic range and/or sufficient accessible conductance states (precision) of weight values^58^. Although voltage controlled analog switching of the present device could help to access various resistance states, yet it is widely acknowledged that an ON/OFF ratio of 10 or above is required for better differentiation between the potentiation and depression states, which is important for accurate synaptic weight updates. In an array level integration with memristors, a lower ON/OFF ratio suffers from scalability issues as the minimum read margin cannot be satisfied in a large array. Therefore, it needs to be further pursued to improve the ratio with material optimization, or process parameter optimization such as, deposition parameters, annealing conditions.

Energy consumption of electronic synaptic devices in each operation is one of key parameters to have a promising application in imitating synapses. The reported energy consumption varies between ranges of 0.06 fJ to 800 pJ per synaptic event^53,59–62^. By considering the pulse width time, response current, and pulse amplitude, the energy consumption of our device was calculated to be 1.37 × 10^–10^ J (137 pJ) per synaptic event for a 20 µs pulse at a reading voltage of 0.5 V. Nevertheless, the amount energy required per synaptic operation is also affected by the pulse amplitude and pulse width, which could be optimized furthermore for achieving an ultra-low switching operation.

## Conclusion

The highly linear, symmetric, and wide dynamic range of conductance change characteristics were demonstrated by optimizing update operation conditions to control the filament formation in memristive Ti/HfO_2_/oxygen-deficient-HfO_2−x_/Pt devices, which contains oxide/suboxide bilayer switching oxide with different oxygen stoichiometry. The XPS analysis confirmed the different concentrations of oxygen vacancies in each layer which assists in controlled filament formation and improved device stability, particularly for gradual conductance change for analog synapse applications. The bilayer devices showed an excellent low voltage operation with analog conductance modulation. The synaptic characteristics of the device could be further improved using various pulse conditions. The conductance change for synaptic weight update was optimized to be highly gradual, linear, and symmetric with a wide dynamic range and good endurance by reducing pulse steps in ISPP operations for potentiation and depression behaviors. Furthermore, the neural network learning simulation results by using a three-layer MLP neural network on the MNIST handwritten digits dataset exhibited the learning accuracy of ~ 80% on average. These results demonstrate the high performance of synaptic device composed of the oxide/suboxide homojunction hafnium oxide layer by optimizing operation conditions for future hardware-based neuromorphic computing systems.

## Experimental method

The artificial synaptic memristive devices have a structure of Ti/HfO_2_/HfO_2−x_/Pt with Ti and Pt as top and bottom electrode, respectively, as shown in Fig. 1. The switching layer of the device is composed of two layers of hafnium oxides with different stoichiometries: an upper stoichiometric HfO_2_ layer and a lower oxygen-deficient HfO_2−x_ layer. First, a 100 nm thick Pt bottom electrode with a Ti (30 nm) adhesion layer was deposited on SiO_2_/Si substrate by e-beam evaporation. The two hafnium oxide layers with a thickness of ~ 3 nm each were deposited by ALD system using tetrakis(ethyl-methyl-amino) hafnium (TEMAHf) and water (H_2_O) as precursor and reactant at temperature of 200 °C for stoichiometric HfO_2_ layer and 100 °C for oxygen-deficient HfO_2−x_ layer. Finally, a 70 nm thick Ti top electrode was deposited by e-beam evaporation using a shadow mask with a diameter of 100 µm. Subsequently, the devices were annealed at 300 °C for 1 h in air.

The DC voltage sweep measurements were performed using Agilent 4156B semiconductor parameter analyzer at room temperature. The pulse measurements were carried out using a Keithley 4200-SCS semiconductor characterization system combined with a 4225-PMU module. During all electrical characterization, positive bias voltage was applied to the top Ti electrode while the bottom Pt was grounded. The microstructure characterization was carried out using JEOL JEM-ARM300F transmission electron microscope (TEM) for which the cross-sectional TEM sample was prepared by focused ion beam method (Quanta 3D FEG). To investigate the chemical bonding states and oxygen distribution in the bilayer device, X-ray photoelectron spectroscopy (XPS) analysis was carried out. XPS depth profile of bilayer structure was obtained by Ar ion etching.

To evaluate the pattern recognition capability of the device, neural network simulation was performed using the well-known Modified National Institute of Standards and Technology (MNIST) handwritten dataset. Using a comprehensive platform named, NeuroSim + , a supervised artificial neural network learning simulator was used for on-chip training^50^. To examine learning accuracy, synaptic parameters such as the non-linearity, conductance level and cycle-to-cycle variation of the device were input to the simulator.

## Supplementary Information

Supplementary Figures.

## Data Availability

The datasets used and/or analyzed during the current study are available from the corresponding author on reasonable request.
