# Supplementary material for: Linear and symmetric synaptic weight update characteristics by controlling filament geometry in oxide/suboxide HfOx bilayer memristive device for neuromorphic computing
Source: Sci Rep. 2023 Jun 13;13:9592. doi: 10.1038/s41598-023-36784-z (PMC10264419; doi:10.1038/s41598-023-36784-z)
Supplement: Supplementary file 1 — Supplementary Figures. [file 41598_2023_36784_MOESM1_ESM.docx]

**Supplementary Information**

**Linear and Symmetric Synaptic Weight Update Characteristics by Controlling Filament Geometry in Oxide/Suboxide HfO_x_ Bilayer Memristive Device for Neuromorphic Computing**

Dwipak Prasad Sahu^a^, Kitae Park^b^_,_ Peter Hayoung Chung^b^_,_ Jimin Han^a^_,_

and Tae-Sik Yoon^a,b*^


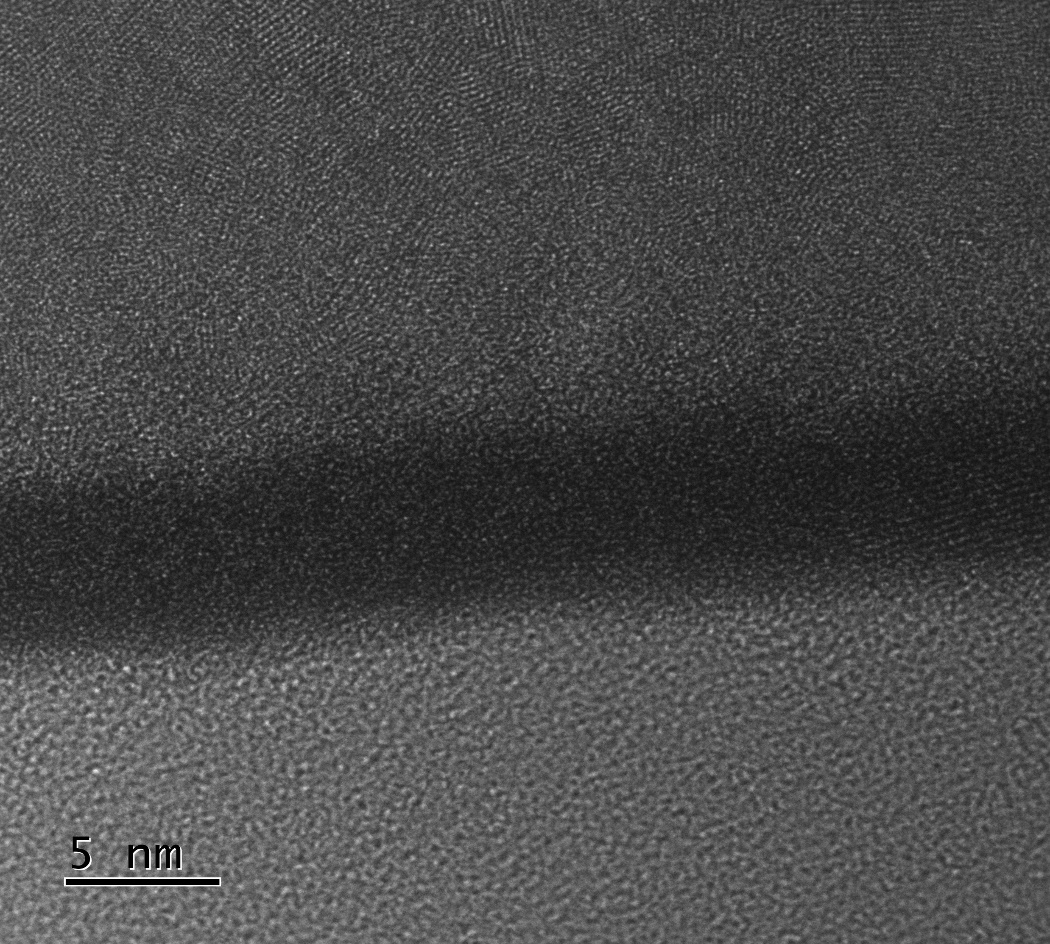


HfO_2_/ HfO_2-x_

Ti

SiO_2_

**Figure S1**. Cross-sectional transmission electron microscopy (TEM) image of the HfO_2_/HfO_2-x_ switching layers deposited on SiO_2_ substrate for a clear distinction.

**Figure S2**. I-V characteristics curves of Ti/HfO_2_/HfO_2-x_/Pt devices for 300 sweep cycles.

**Figure S3:** Endurance performance of the Ti/HfO_2_/HfO_2-x_/Pt device at a read voltage of 0.5V after set and reset at 1V and -1.2V, respectively.

**Figure S4**: XPS spectra of Hf 4f peak evolution over the etching time.

**Figure S5**: XPS spectra of O 1s peak evolution over the etching time.

**Figure S6:** **(a)** Non-linearity of synaptic weight update from Ti/HfO_2_/HfO_2-x_/Pt bilayer
memristor by using identical pulse amplitude of ±1.5V. **(b)** Repetitive conductance modulation with a pulse amplitude of ±1.5V, a pulse width of 10 µs, and a pulse Interval time of 100 µs. The current was read at 0.5V.
